# Advanced Management of Acute Intermittent Porphyria: The Role of Givosiran Therapy in Improving Long‐Term Outcomes‐A Case Study

**DOI:** 10.1002/ccr3.73012

**Published:** 2026-07-05

**Authors:** Natália Rebeca Alves de Araújo Karpejany, Jose Tiburcio do Monte Neto, Ester Miranda Pereira, Isabel Andrea Ferreira Carvalho, Rafael Melo Santos de Serpa Brandão, Adalberto Socorro da Silva, Tiberio Silva Borges dos Santos, Semiramis Jamil Hadad do Monte

**Affiliations:** ^1^ Hospital Universitário da Universidade Federal do Piauí Teresina Brazil; ^2^ Laboratório de Imunogenética e Biologia Molecular ‐ Universidade Federal do Piauí Teresina Brazil

**Keywords:** acute intermittent porphyria, gene silencing, givosiran, hemin, hydroxymethylbilane synthase

## Abstract

Acute intermittent porphyria is a rare disorder causing neurotoxic precursor accumulation and severe neurological complications. We report a case progressing to tetraplegia and respiratory failure with delayed diagnosis. Treatment with hemin and givosiran resulted in prevention of attacks and functional recovery, highlighting the importance of early diagnosis and long‐term therapy.

## Key Clinical Message

Early recognition of acute intermittent porphyria is critical to prevent irreversible neurological damage. In severe cases with advanced neuropathy and respiratory failure, long‐term treatment with givosiran may allow significant functional recovery and prevent recurrence, even in the presence of persistent biochemical abnormalities.

**Abbreviations**
AHP: Acute Hepatic Porphyrias
AIP: Acute Intermittent Porphyria
ALA: Aminolevulinic Acid
ALAS1: Delta‐Aminolevulinic Acid Synthase 1
HMBS: Hydroxymethylbilane Synthase
NGS: Next‐Generation Sequencing
NMD: Nonsense‐Mediated mRNA Decay
PBG: Porphobilinogen
siRNA: Small Interfering RNA
SUS: Sistema Único de Saúde (Brazilian Unified Health System)

## 1 Introduction

Porphyrias comprise a heterogeneous group of rare metabolic disorders caused by partial deficiency of one of the eight enzymes involved in the heme biosynthesis pathway [1]. Depending on the predominant site of enzyme deficiency, porphyrias are classically classified as erythropoietic or hepatic [2, 3]. From a clinical standpoint, they are further categorized into acute and cutaneous forms [4]. In acute porphyrias, impaired enzymatic activity results in hepatic overproduction and accumulation of neurotoxic heme precursors, leading to potentially severe neurovisceral manifestations [5]. In contrast, cutaneous porphyrias are characterized by the accumulation of phototoxic porphyrins, resulting primarily in photosensitivity [4, 5].

Acute intermittent porphyria (AIP) is the most common and clinically severe subtype among the acute hepatic porphyrias [6]. It is caused by pathogenic variants in the hydroxymethylbilane synthase (HMBS) gene, leading to reduced enzymatic activity in the third step of heme biosynthesis [7]. The resulting deficiency triggers compensatory upregulation of hepatic aminolevulinic acid synthase 1 (ALAS1), the rate‐limiting enzyme of the pathway, with consequent accumulation of aminolevulinic acid (ALA) and porphobilinogen (PBG) [8]. These intermediates exert direct neurotoxic effects and are central to the pathophysiology of acute attacks [1, 8, 9].

Clinically, AIP manifests as acute neurovisceral crises characterized by severe abdominal pain, autonomic instability (including tachycardia and hypertension), neuropsychiatric symptoms, and progressive motor neuropathy [9, 10]. In severe cases, this neuropathy may evolve into flaccid quadriplegia and respiratory failure, requiring prolonged intensive care support [10]. Disease penetrance is incomplete and highly variable, influenced by genetic modifiers and environmental factors [1, 9]. Acute attacks are typically precipitated by conditions that induce hepatic ALAS1 activity, such as exposure to porphyrinogenic medications, hormonal fluctuations, fasting, infection, or physiological stress [7, 9].

The nonspecific nature of early AIP manifestations frequently leads to diagnostic delay, particularly in emergency settings, where abdominal pain and neurological symptoms are more commonly attributed to prevalent conditions [6, 11, 12]. Delayed recognition and treatment are strongly associated with increased risk of irreversible neurological damage and long‐term disability. Therefore, rapid biochemical confirmation during an acute crisis is essential for timely initiation of disease‐specific therapy [10, 13].

Traditional management of acute attacks relies on carbohydrate loading and intravenous hemin administration to suppress ALAS1 induction and reduce the synthesis of neurotoxic intermediates [8, 10]. While these interventions are effective in aborting acute crises, they do not prevent recurrence in patients with high disease activity or chronic biochemical overproduction. Consequently, individuals with recurrent or severe AIP may develop a chronic disease course with substantial impairment in quality of life [14].

The therapeutic landscape of AIP has been substantially transformed by the development of givosiran, a subcutaneously administered small interfering RNA that selectively silences hepatic ALAS1 expression [14, 15]. By targeting the upstream driver of heme precursor overproduction, givosiran provides sustained biochemical suppression and reduces the frequency and severity of acute attacks [14]. This therapeutic approach represents a paradigm shift from episodic crisis management toward long‐term disease modification.

## 2 Case History/Examination

A 28‐year‐old previously healthy man, employed as a dairy factory worker, was admitted following a motor vehicle accident. Initial evaluation revealed only superficial abrasions, with no loss of consciousness or neurological deficits. He was discharged from the emergency department with prescribed analgesic medications.

Three days later, the patient developed severe, diffuse abdominal pain accompanied by constipation, nausea, and vomiting, which were refractory to symptomatic treatment. Over the subsequent days, he progressed to urinary retention and descending muscle weakness. Despite multiple medical evaluations over a 10‐day period, the underlying cause remained unidentified. On the tenth day after symptom onset, he experienced a generalized tonic‐clonic seizure and was transferred to a tertiary care center for further investigation.

Upon admission, the patient presented with marked autonomic instability, including severe hypertension (180/110 mmHg) and sinus tachycardia (120 beats per minute), as well as altered mental status. His urine was noted to be markedly dark. Two days after admission, corresponding to 12 days after the triggering trauma, acute porphyria was clinically suspected based on the constellation of neurovisceral symptoms.

## 3 Differential Diagnosis, Investigations and Treatment

Immediate biochemical screening revealed a strongly positive qualitative urinary porphobilinogen (PBG) test, along with markedly elevated urinary ALA (35 mg/g creatinine), establishing the diagnosis of AIP.

Following biochemical confirmation, treatment was initiated with high‐dose glucose supplementation (300–500 g/day), administered via enteral and parenteral routes. Intravenous hemin therapy was started 25 days after biochemical diagnosis, at a maximum dose of 4 mg/kg/day for four consecutive days, selected due to the life‐threatening severity of the presentation. At that time, the patient exhibited flaccid tetraplegia, urinary retention, generalized hypoesthesia, and acute respiratory failure requiring invasive mechanical ventilation.

After the initial hemin cycle, partial clinical improvement was observed, including modest gains in muscle strength and ventilatory parameters. However, 49 days after first cycle, clinical and laboratory deterioration occurred, likely triggered by an intercurrent infection, characterized by worsening respiratory function, increased limb weakness, intensified biochemical activity, and rising ALA levels (39.58 mg/g creatinine). A second cycle of hemin was administered, resulting in gradual stabilization.

Management of autonomic dysfunction required beta‐blockers for persistent tachycardia and multiple antihypertensive agents for refractory hypertension. Severe abdominal pain was controlled with opioid analgesia. Over the course of five months of hospitalization, the patient demonstrated progressive neurological recovery, with restoration of cervical and truncal mobility, normalization of bowel and bladder function, improved hemodynamic stability, and successful weaning to minimal pressure‐support ventilation.

## 4 Conclusion and Results (Outcome and Follow‐Up)

At hospital discharge, the patient remained neurologically impaired, presenting with paraplegia of the lower limbs, paresis of the upper limbs, and hypoesthesia in all four extremities. He was able to maintain trunk posture independently and remained tracheostomized but breathing spontaneously on room air. Qualitative biochemical testing for porphobilinogen remained positive.

Long‐term prophylactic therapy with givosiran was initiated after discharge at a dose of 2.5 mg/kg administered subcutaneously on a monthly basis. At 12 months of clinical follow‐up, the patient had experienced no further acute porphyric attacks. Neurological examination demonstrated significant functional improvement, including the ability to ambulate with assistance. Motor strength was graded according to the Medical Research Council (MRC) scale (where 0 indicates no muscle contraction and 5 indicates normal strength) as V proximally and III distally (active movement against gravity but not resistance) in the upper extremities, and V proximally and I distally (flicker or trace of contraction) in the lower extremities. Deep tendon reflexes remained globally absent, consistent with residual severe axonal neuropathy.

Table 1 shows the local biochemical monitoring starting from the seventh dose of givosiran. Throughout the entire period, from hospital admission onward, qualitative urinary PBG testing remained positive. From a quantitative perspective, normalization of ALA levels was observed, whereas PBG levels remained elevated. Quantitative baseline and early post‐discharge monitoring focused predominantly on ALA levels due to initial regional laboratory limitations in performing automated sequential quantitative PBG testing, which was standardized from the seventh month onward.

**TABLE 1:** Monthly urinary levels of ALA and PBG after 6 months of starting givosiran therapy, including pre‐treatment baseline levels.

Month	ALAu (mg/g creatinine) n.r. ≤ 3.6	PBGu mg/g creatinine n.r. ≤ 2.2
Basal (pre‐treatment)	39.58	Positive^a^
7°month	3.41	6.57
8°month	1.10	18.75
9°month	4.94	18.97
10°month	0.85	11.68
11°month	0.78	18.24
12°month	0.99	8.10

*Note:* Quantitative analysis was conducted using the ClinEasy kit (RECIPE). Values are expressed in milligrams of analyte per gram of Creatinine. Upper reference limits: ALA ≤ 3.6 mg/g Creatinine; PBG ≤ 2.2 mg/g Creatinine.

Abbreviations: ALAu, urinary aminolevulinic acid; n.r., normal reference range; PBGu, urinary porphobilinogen.

^a^ Quantitative baseline and early post‐discharge monitoring focused predominantly on ALA levels due to initial regional laboratory limitations in performing automated sequential quantitative PBG testing, which was standardized from the seventh month onward.

**FIGURE 1:**
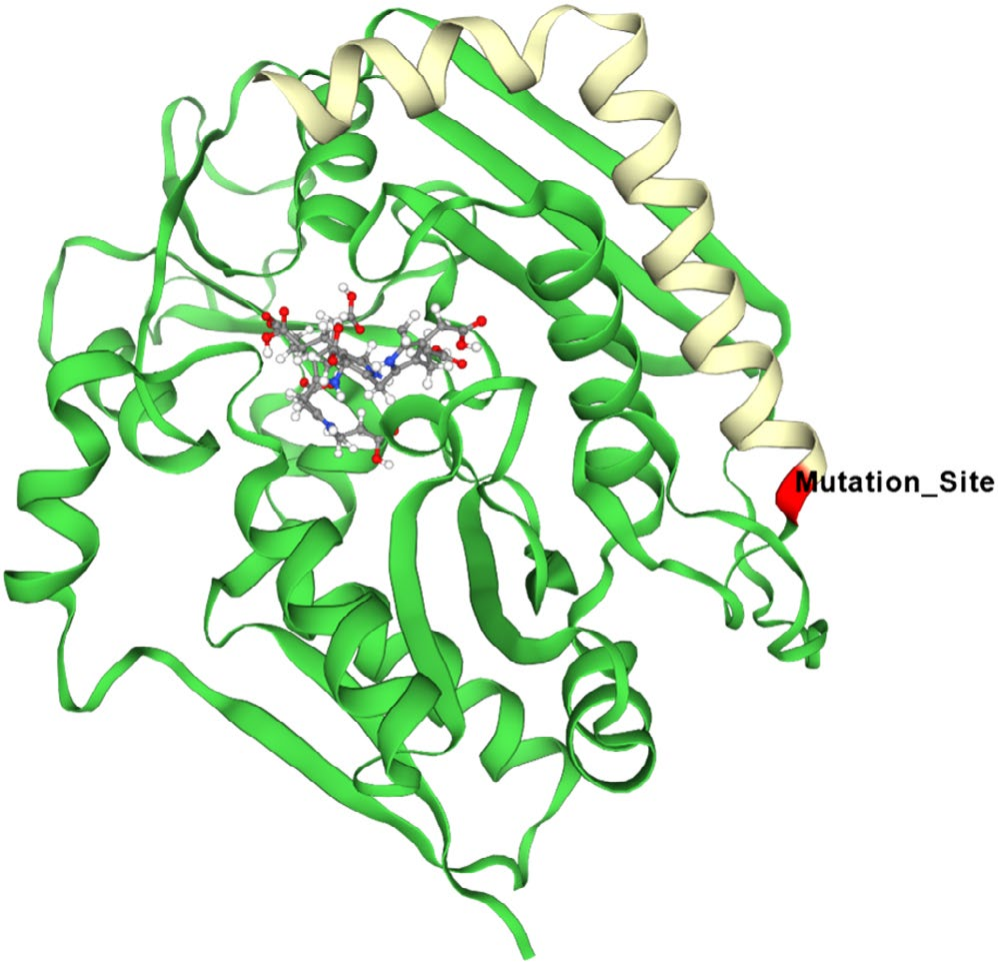
Structure of human HMBS modeled from the crystal structure (PDB ID: 5M6R). HMBS is shown in green, the R325 mutation site is shown in red, and the 36‐residue lost region due to the stop codon activated at position 325 is shown in light yellow.

Genetic analysis of the region of interest was performed using next‐generation sequencing. This analysis identified a pathogenic nonsense variant located in exon 15 of the *HMBS* gene, c.973C > T (p.Arg325*), in heterozygosity, in both the patient and his mother. This mutation and its structural impact on the protein are illustrated in Figure 1.

Following her son's diagnosis, the mother reported a mild episode of abdominal pain associated with positive biochemical findings, including elevated urinary PBG and ALA levels (10.17 mg/g and 6.40 mg/g, respectively). She received counseling regarding avoidance of precipitating factors and remains asymptomatic under clinical surveillance. While the patient required full therapeutic intervention with hemin and givosiran, genetic confirmation in the mother was essential for preventive management and family counseling.

## 5 Discussion

This case illustrates the severe end of the clinical spectrum of AIP, characterized by rapid progression from nonspecific abdominal symptoms to profound motor neuropathy and life‐threatening respiratory failure. Such presentations remain diagnostically challenging, particularly in emergency settings, where gastrointestinal complaints and neurological deficits are more commonly attributed to prevalent conditions such as Guillain‐Barré syndrome, spinal cord injury, or metabolic encephalopathies [7, 12]. In this patient, the temporal association with trauma further contributed to diagnostic uncertainty, resulting in a substantial delay between symptom onset and disease recognition.

Delayed diagnosis is a well‐recognized determinant of poor neurological outcomes in AIP. The accumulation of neurotoxic heme precursors, particularly ALA and PBG, exerts direct effects on the peripheral and autonomic nervous systems, leading to axonal degeneration that may become irreversible if exposure is prolonged [1, 9]. In the present case, clinical suspicion arose 12 days after the triggering event, and disease‐specific therapy was initiated even later, a sequence consistent with reports from international case series describing prolonged diagnostic intervals and severe residual deficits [10, 13].

Rapid biochemical confirmation during an acute neurovisceral crisis is therefore critical. In clinical practice, bedside qualitative screening based on Ehrlich's reaction plays a pivotal role in enabling early therapeutic decisions [14]. Strictly speaking, Ehrlich's reagent refers to a chemical reaction rather than a diagnostic test per se. In the clinical laboratory context, however, this reaction underpins qualitative urinary porphobilinogen testing, which includes phase separation steps to distinguish porphobilinogen from interfering substances [11, 14]. When markedly positive during an acute attack, this qualitative assessment can serve as a vital tool in emergency settings. However, it is imperative to emphasize that qualitative screening should not be used to justify the initiation of specific therapies like hemin in stable patients, given the potential for false‐positive results and the well‐documented phenomenon of ‘chronic high excretors’ (who maintain high excretion for years without active crises). In stable or undiagnosed patients presenting with pain or mild neurological symptoms, a positive qualitative test should prompt the elimination of porphyrinogenic drugs, initiation of intravenous carbohydrates, symptomatic management, and urgent confirmatory quantitative testing. In our exceptional case, however, the patient presented with rapidly progressive, life‐threatening manifestations, including flaccid tetraplegia and respiratory failure, which clinically justified the immediate initiation of hemin while quantitative validation was pending [8, 10, 11].

Quantitative measurement of urinary PBG and ALA complements testing by allowing confirmation, monitoring of biochemical activity, and phenotypic characterization over time [9, 14]. In this case, the availability of both qualitative and quantitative assays was essential not only for establishing the diagnosis but also for documenting persistent biochemical activity after clinical stabilization. The consolidation of such diagnostic capacity in regional reference centers is a critical step toward reducing inequities in access to care and minimizing preventable neurological injury [11].

Acute management with carbohydrate loading and intravenous hemin remains the cornerstone of treatment for AIP crises, as these interventions suppress hepatic ALAS1 induction and reduce precursor synthesis [8, 10]. Nonetheless, this case underscores the limitations of acute therapy alone. Despite clinical stabilization, the patient was discharged with severe residual neuropathy and persistent biochemical activity, reflecting a transition from episodic disease to a chronic active phenotype [12, 15].

Long‐term prophylaxis with givosiran has fundamentally altered the management of patients with recurrent or severe AIP. By selectively silencing hepatic ALAS1 mRNA, givosiran targets the upstream driver of precursor overproduction, resulting in sustained suppression of ALA and PBG synthesis [16, 17]. Clinical trials and long‐term extension studies have demonstrated marked reductions in attack frequency, decreased reliance on hemin, and significant improvements in quality of life [15, 17]. The present case aligns with these findings, as 1 year of givosiran therapy was associated with complete prevention of further acute attacks and meaningful functional neurological recovery [18].

Importantly, this patient remained a chronic high excretor of porphobilinogen despite clinical remission. Persistent biochemical activity in the absence of overt attacks has been associated with an increased risk of symptomatic recurrence and long‐term complications. Furthermore, chronic high excretors carry a significantly higher risk for developing hepatocellular carcinoma (HCC) compared to individuals with normal excretion, necessitating rigorous, long‐term surveillance via annual liver ultrasonography and alpha‐fetoprotein monitoring [6, 8, 15, 18]. In this context, continued givosiran therapy is justified as a preventive strategy aimed at maintaining disease control and reducing the risk of future neurological deterioration.

Genetic confirmation of the pathogenic variant in the HMBS gene was not required for acute diagnosis or therapeutic decision‐making but proved essential for family screening and preventive care [1, 7]. Identification of the same mutation in the patient's mother enabled targeted counseling, avoidance of precipitating factors, and clinical surveillance, illustrating the importance of molecular diagnosis in extending the benefits of care beyond the index case.

Currently, the UniProt database [19] contains a total of 473 variants in the HMBS gene, of which 160 are considered pathogenic or likely pathogenic [20]. The mutation found here (c.973C > T) was first described by Petersen et al. [21] in a Danish family. After its discovery, other studies identified this mutation in AIP patients around the world, including Brazil [11, 20, 22].

The c.973C > T (p.Arg325*) mutation corresponds to a nonsense variant in the HMBS gene that introduces a premature stop codon, leading to the loss of 36 amino acids from the C‐terminal region of the protein [20]. This alteration results in the production of a truncated protein or, more likely, degradation of the mutant transcript through the nonsense‐mediated mRNA decay (NMD) pathway, culminating in significant loss of enzymatic function [23].

Overall, this case exemplifies how delayed recognition of AIP can lead to catastrophic neurological outcomes, while timely biochemical diagnosis and access to disease‐modifying therapy can substantially alter the disease course. The integration of rapid qualitative screening, confirmatory quantitative testing, acute hemin therapy, and long‐term prophylaxis with givosiran represents a comprehensive approach capable of improving both survival and long‐term functional prognosis in patients with severe AIP.

## 6 Conclusion

This report describes a severe presentation of acute intermittent porphyria (AIP) caused by a pathogenic HMBS nonsense variant, leading to rapidly progressive motor neuropathy and life‐threatening respiratory failure after exposure to precipitating factors. It highlights the critical impact of delayed diagnosis and the need for high clinical suspicion in patients with acute abdominal and neurological symptoms. Early clinical suspicion and timely biochemical confirmation are essential for prompt treatment and the prevention of irreversible neurological damage. While intravenous hemin remains the cornerstone of acute management, this case underscores its limitations in patients with chronic active disease. The sustained clinical improvement observed with givosiran therapy, including prevention of new attacks and significant neurological recovery, reinforces the importance of combining early diagnosis with long‐term disease‐modifying strategies to improve outcomes in severe AIP.

## Author Contributions

**Ester Miranda Pereira:** investigation, formal analysis, writing – review and editing. **Adalberto Socorro da Silva:** methodology, investigation, writing – review and editing. **Natália Rebeca Alves de Araújo Karpejany:** conceptualization, investigation, writing – original draft, data curation, resources, project administration. **Rafael Melo Santos de Serpa Brandão:** investigation, methodology, validation, writing – review and editing. **Isabel Andrea Ferreira Carvalho:** investigation, writing – review and editing, resources. **Semiramis Jamil Hadad do Monte:** supervision, conceptualization, investigation, writing – review and editing, formal analysis, project administration. **Jose Tiburcio do Monte Neto:** conceptualization, investigation, supervision, writing – review and editing. **Tiberio Silva Borges dos Santos:** writing – review and editing, investigation, supervision.

## Funding

The authors have nothing to report.

## Ethics Statement

The authors confirm that the approval of an institutional review board was not required for this work. We confirm that we have read the Journal's position on issues involved in ethical publication and affirm that this work is consistent with those guidelines.

## Consent

Written informed consent from the patient was obtained according to journal guidelines.

## Conflicts of Interest

The authors declare no conflicts of interest.

## Data Availability

The data that support the findings of this study are available from the corresponding author upon reasonable request.
